# Thrombus Formation on the Tricuspid Valve After De Vega’s Annuloplasty and Repair of Endocardial Cushion Defect

**DOI:** 10.15171/jcvtr.2014.012

**Published:** 2014-09-30

**Authors:** Süleyman Çağan Efe, Tuba Unkun, Servet İzci, Murat Çap, Ruken Bengi Bakal, Rezzan Deniz Acar, Çetin Geçmen, Emrah Erdoğan, Nihal Özdemir

**Affiliations:** Department of Cardiology, Kosuyolu Kartal Heart Training and Research Hospital, Istanbul, Turkey

**Keywords:** Congenital Heart Defects, Tricuspid Valve, Mass

## Abstract

Endocardial cushion defect (ECD) can be partial (with two distinct valves) or complete (only one atrioventricular valve), and surgical therapy is usually required. The optimal surgical technique is controversial but De Vega’s annuloplasty is widely performed. Tricuspid valve thrombosis are rarely seen after surgery. We present a 39-year-old male patient with tricuspid valve thrombosis after De Vega’s annuloplasty without the use of a ring.

## Case report

A 39-year-old male was referred to our hospital because of a cardiac murmur in his physical examination. Echocardiogram was performed and ostium primum atrial septum defect (ASD) and inlet type ventricular septum defect (VSD) were determined. On transesophageal echocardiography (TEE) , a cleft in posterior mitral leaflet was seen as well.

The patient was referred for surgery in our center. Repair of both ASD and VSD, mitral cleft repair, and De Vega’s annuloplasty was performed. The post-operative course was uneventful, and he was discharged on metoprolol and acetylsalicylic acid (ASA). On his 6-month follow-up echocardiogram, because of a 2 × 1.2 cm mass was found on his septal leaflet of tricuspid valve (Figure 1-A; Supplemantary video 1), TEE was performed . A mass of 2.1 × 1.5 cm with a smooth border originating from septal and anterior leaflets was seen (Figure 1B-C ) on TEE. There was no defect on atrial and ventricular septum.

Figure 1A) Transthoracic echocardiographic image showing a 2 x 1.2 cm mass on septal leaflet of tricuspid valve (LV, left ventricle; RV, right ventricle; RA, right atrium). B) Transesophageal echocardiographic image showing a 2.1 x 1.5 cm mass with a smooth border originating from septal and anterior leaflets of tricuspid valve (LV, left ventricle; RV, right ventricle; RA, right atrium). C)On 3D Transesophageal echocardiography, the mass on septal leaflet of tricuspid valve was seen. D) Postoperative image of resected material.

**A:**
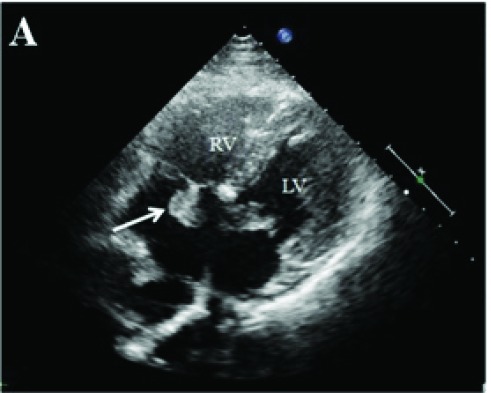

**B:**
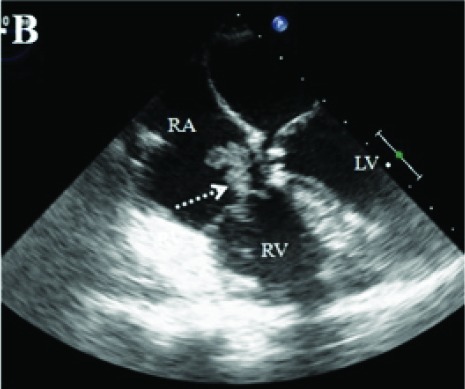

**C:**
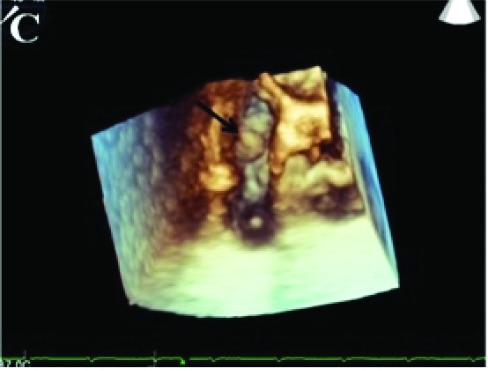

**D:**
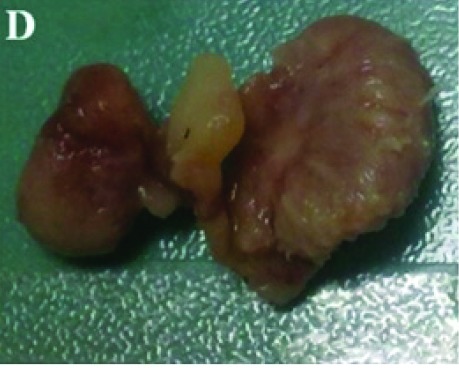

Potential causes for thrombosis were examined; blood analysis showed activated partial thromboplastin time of 24 s (normal range: 20-40 s), prothrombin time of 13 s (normal range: 10-14 s) and international normalized ratio (INR) of 1.2 Lupus anticoagulant was negative and anti-cardiolipin antibodies were low. He had no history of hypercoagulopathy or deep vein thrombosis.

For prevention of embolism and exclusion of tumor, surgical operation was performed. The excised specimen was reported as an organized thrombus (Figure 1-D), therefore, anticoagulation with warfarin was prescribed for 6 months after the operation.

## Discussion

The common atrioventricular canal was divided into left and right by the fusion of superior and inferior endocardial cushions. A defect in this stage of development result in ostium primum ASD, inlet type VSD and structural defects in atrioventricular valves. Endocardial cushion defect (ECD) can be partial (with two distinct valves) or complete (only one atrioventricular valve), and surgical correction is almost always required. Tricuspid valve repair is indicated in patients who have moderate to severe tricuspid regurgitation and undergoing cardiac surgery. The optimal surgical technique is controversial but De Vega’s annuloplasty was widely performed. However, in patient with severe annular dilatation and pulmonary hypertension recurrence is common with De Vega’s procedure and ring annuloplasty is preferred.^1^

Tricuspid valve thrombosis are quite rare and can be examined in 3 groups; 1) Patients with antiphospholipid syndromes, 2) Patients with structural defects and 3) Idiopathic thrombus formation without any coagulopathy or structural defect. Tricuspid valve thrombus in patients with antiphospholipid syndrome can present with symptoms of tricuspid stenosis.^2^ Thrombus formation in patient with structural defect is rather rare. Konishi et al reported a case of organized thrombus of tricuspid valve in a patient with VSD and tricuspid pouch.

Stagnation of blood around the septal leaflet pouch was thought to be responsible for thrombus formation.^3^ Mario et al reported another case of tricuspid thrombus in a patient with mild hyperhomocysteinemia who underwent tricuspid ring annuloplasty and ASD repair.^4^ Moreover, tricuspid valve thrombosis was reported in two separate cases with no known cardiac anomaly or coagulopathy. But the origin of thrombus was found to be deep veins.^5,6^

We report a rare case with a tricuspid thrombus formation after De Vega’s annuloplasty without the use of a ring. Although right-sided prosthetic materials have a higher incidence of thrombotic complication, postoperative antithrombotic therapy is not advised after tricuspid valve repair in current practice guidelines. Therefore, the anticoagulant therapy was not administered to this patient. However, it has been reported that the continued use of anti-aggregant therapy should be considered in patients with tricuspid repair after surgery.^7^ Our patient had quitted ASA treatment 3 months after the operation, and the thrombus formation might be precipitated by the cessation of anti-aggregant therapy. In our case, the thrombus was larger than 10 mm and surgical treatment was preferred. In smaller thrombus, follow-up with anticoagulant or anti-aggregant medication may be considered.^7^ In literature, thrombus management was usually done by anticoagulant therapy, and no evidence was found for appropriate use of antithrombotic and anticoagulant regimes. The anticoagulation for at least 3 months is recommended for the patients with venous thromboembolism but, if irreversible risk factors exists, extended anticoagulant therapy should be recommended.^8^ We reevaluated our patient 6 months after surgery with TEE. There was no new thrombus formation, therefore we medicated our patient with ASA instead of warfarin as a long term treatment. The patient had no more events after one-year follow-up.

## Ethical issues

The study was approval by the Local Ethics Committee.

## Competing interests

Authors declare no conflict of interests in this study.

## Supplementary materials

Supplementary video 1:
Transthoracic subcostal view showing a mass on septal leaflet of tricuspid valve.
